# Prevalence and impact of orofacial pain in older adults with dementia: a systematic review and meta-analysis

**DOI:** 10.1590/2317-1782/e20250380en

**Published:** 2026-07-31

**Authors:** Mylena de Abreu Cardoso, Kaliane Rodrigues da Cruz, Rayane Délcia da Silva, Karinna Veríssimo Meira Taveira, José Stechman-Neto, Cristiano Miranda de Araujo, Thalita de Paris Matos Bronholo, Angela Graciela Deliga Schroder

**Affiliations:** 1 Faculdade de Odontologia, Universidade Tuiuti do Paraná – UTP - Curitiba (PR), Brasil.; 2 Programa de Pós-graduação em Odontologia, Universidade Tuiuti do Paraná – UTP - Curitiba (PR), Brasil.; 3 Programa de Pós-graduação em Distúrbios da Comunicação, Universidade Tuiuti do Paraná – UTP - Curitiba (PR), Brasil.; 4 Programa de Pós-graduação em Saúde da Comunicação Humana, Universidade Tuiuti do Paraná – UTP - Curitiba (PR), Brasil.; 5 Deparmento de Morfologia, Universidade Federal do Rio Grande do Norte – UFRN - Natal (RN), Brasil.; 6 Deparmento de Odontologia Restauradora, Universidade Federal do Paraná – UFPR - Curitiba (PR), Brasil.

**Keywords:** Dementia, Facial Pain, Older Adults, Senile Paranoid Dementia, Orofacial Pain

## Abstract

**Purpose:**

This systematic review and meta-analysis aimed to determine the prevalence and impact of orofacial pain among older adults with dementia.

**Research strategies:**

Observational studies were retrieved from EMBASE, LILACS, PubMed/MEDLINE, Scopus, Cochrane, Livivo, and Web of Science databases, in addition to gray literature. The search included studies without language or date restrictions.

**Selection criteria:**

Eligible studies included participants aged 60 years or older with any type of dementia, reporting the presence, frequency, or characteristics of orofacial pain. Case reports, reviews, and experimental animal studies were excluded.

**Data analysis:**

Data were extracted independently by calibrated reviewers. Meta-analyses were performed using RStudio software, calculating pooled prevalence rates with 95% confidence intervals. Heterogeneity was assessed using the I^2^ statistic.

**Results:**

The overall prevalence of orofacial pain among older adults with dementia was 19%, regardless of dementia type. Associated factors included poor oral hygiene, presence of natural dentition, and xerostomia. Communication difficulties, particularly in non-verbal patients, represented the main diagnostic barrier.

**Conclusion:**

Approximately one in five older adults with dementia experiences orofacial pain, which significantly affects quality of life. However, underdiagnosis remains common due to cognitive and communicative limitations. Improving professional training, multidisciplinary collaboration, and public health strategies focused on oral healthcare in long-term care facilities are essential to enhance pain detection and management.

## INTRODUCTION

Pain is defined as an unpleasant sensory and emotional experience associated with, or resembling that associated with, actual or potential tissue damage^(1)^. Orofacial pain refers to any pain related to the maxillomandibular complex, which can be of odontogenic origin, such as dental, or non-odontogenic pain, like musculoskeletal pain^(2)^. Cognitive decline may be associated with a higher prevalence of orofacial pain. A study on older individuals diagnosed with Alzheimer's disease found that 20.7% of participants had experienced myofascial or periodontal pain^(3)^.

Orofacial pain can significantly impact patients' quality of life, particularly among older adults with dementia, where recognizing pain is difficult. In non-verbal individuals, the challenge of communicating pain may worsen their condition, leading to increased psychological suffering. This can create a cycle where pain intensifies psychological distress, which then heightens pain perception^(4,5)^.

Dementia, as defined by the Diagnostic and Statistical Manual of Mental Disorders, is a neurocognitive disorder that affects specific cognitive areas, including memory, learning, language, executive functions, and social judgment. These impairments interfere with interpersonal and occupational functioning, leading to a significant decline from previous levels^(6)^.

About half of older individuals with dementia experience pain and require pain management relievers^(7)^. However, the literature does not show a clear consensus on this prevalence, which complicates the dental management of these patients^(7)^. Therefore, understanding the connection between orofacial pain and dementia is crucial for providing appropriate care and improving the quality of life for vulnerable patients. Two systematic reviews have been published to assess the prevalence of orofacial pain in older individuals with dementia, showing that orofacial pain is more common among institutionalized older adults, who often have poorer overall health^(8,9)^. However, these reviews focused only on hard tissues^(8)^ and included not only dementia patients but also individuals with mild cognitive impairment^(9)^.

This study aimed to assess the prevalence of orofacial pain in older adults with dementia through a systematic review and meta-analysis. It included all types of orofacial pain without restrictions based on region or tissue type and focused exclusively on patients with dementia.

## METHODS

### Protocol and registration

The systematic review was developed in accordance with the Preferred Reporting Items for Systematic Reviews and Meta-Analyses (PRISMA 2020 Statement) guidelines^(10)^. The research protocol was registered on the International Prospective Register of Systematic Reviews – York University Center for Commentary and Dissemination (PROSPERO) under registration number CRD42024530078.

### Eligibility criteria

The PECOS framework established the eligibility criteria to address the focused question: “What is the prevalence of orofacial pain in older individuals with dementia?”

- **P** = Population: Older individuals
- **E** = Exposure: Dementia
- **C** = Comparison: Not applicable (prevalence study)
- **O** = Outcome: Prevalence of orofacial pain
- **S** = Study design: Observational studies

### Inclusion criteria

Cross-sectional, cohort, and case-control studies that report the frequency of orofacial pain in dementia patients were included. Studies focusing on older populations, without restrictions based on gender, ethnicity, region, or facial tissue type, were eligible.

### Exclusion criteria

Studies were excluded if they did not report outcomes related to orofacial pain in older adults (Appendix 1). They were also excluded if they did not report orofacial pain or lacked prevalence data, even after contacting the authors. Reviews, systematic reviews, expert opinions, in vitro or animal studies, books or book chapters, conference abstracts, case reports, and series were also excluded.

### Search strategy

A search strategy combining keywords and truncations was applied to the following electronic databases: EMBASE, LILACS, PubMed/Medline, Scopus, Cochrane, Livivo, and Web of Science (Appendix 2). Gray literature was researched using Google Scholar and ProQuest Dissertations and Theses. Searches were conducted in June 2023 and updated in September 2025.

Additionally, manual searches were conducted in the references of included articles using Citation Chaser, a tool that retrieves references of included articles and articles citing them. An expert was also consulted to identify potentially eligible studies. References retrieved through the search strategy were managed, and duplicates were removed using EndNote® software (EndNote® X7 Thomson Reuters).

### Study selection

The selection process was conducted in two phases. First, two reviewers independently assessed the titles and abstracts of all references (Phase 1). Studies that did not meet the predefined eligibility criteria were excluded. In Phase 2, the same reviewers independently read the full texts of selected articles, applying the same eligibility criteria. A third reviewer resolved disagreements.

To ensure impartiality, both phases were conducted using Rayyan®^(11)^. A preliminary literature search was conducted to calibrate the

selection process, and inter-reviewer agreement was calculated using the Kappa coefficient. Selection began only after agreement levels exceeded 0.8.

### Data extraction

Two independent reviewers extracted data from the included studies. A third reviewer resolved disagreements. Extracted data included study characteristics (authors, publication year, country, and design), population characteristics (sample size, sex, and age), presence and prevalence of dementia and orofacial pain, and type of reported pain. Absolute or percentage frequency values and sample sizes were also extracted. Only the first period was considered for data collection for studies with multiple follow-up periods. If data were incomplete, up to three attempts were made to contact the authors for additional information. Articles with no response were excluded from the study's quantitative synthesis.

### Risk of bias

The methodological quality of the selected studies was evaluated using the Joanna Briggs Institute Critical Appraisal Tool for cross-sectional, cohort, and case-control studies^(12,13)^. Two reviewers independently assessed the risk of bias, categorizing each criterion as “yes, no, unclear, or not applicable.” A third reviewer resolved any disagreements. Studies were classified as having a “high, moderate, or low” risk of bias based on the percentage of “yes” responses: 0–49% indicated high risk, 50–69% moderate risk, and 70% or more low risk.

### Effect measures

The proportion of the event of interest was calculated to determine the prevalence of orofacial pain, accompanied by a 95% confidence interval (CI).

### Synthesis method

A meta-analysis of proportions was conducted using a random effect model in RStudio (version 1.2.1335; RStudio Inc, Boston, USA). The Freeman-Tukey double arcsine method was applied to normalize the data distribution. The variance was calculated using the DerSimonian-Laird estimator (Tau^2^), and heterogeneity was assessed using the inconsistency index (I^2^). The confidence intervals (95%) were calculated using the Clopper-Pearson method.

## RESULTS

### Study selection

**Figure 1:**
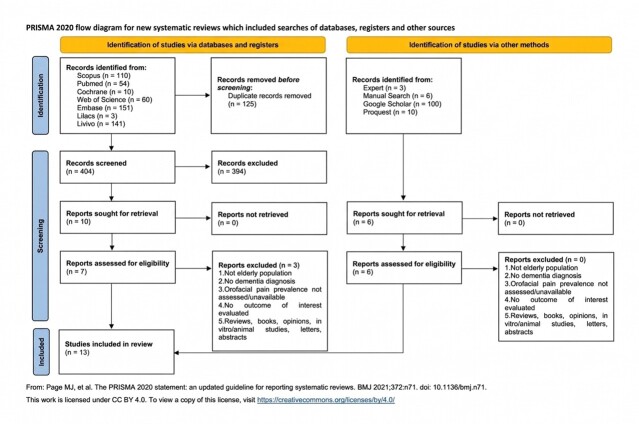
Literature search flowchart and selection criteria

A total of 529 articles were retrieved from database searches, of which 404 remained after duplicate removal (Figure1). Ten articles were selected after the initial screening of titles and abstracts (Phase 1), and seven were included after full-text review (Phase 2). Six additional articles were identified through manual reference searches, bringing the total to 13 articles included in the synthesis.

### Study characteristics

The included studies, published between 2003 and 2020, were conducted in Australia, the United Kingdom, Brazil, Greece, the Netherlands, Switzerland, and France. Nine were cross-sectional, one was a case-control study, and three were cohort studies. Sample sizes ranged from 56 to 5,922 participants, aged 57 to 106. The study characteristics are summarized in Table 1 (Appendix 3).

### Risk of bias in the studies

**Figure 2:**
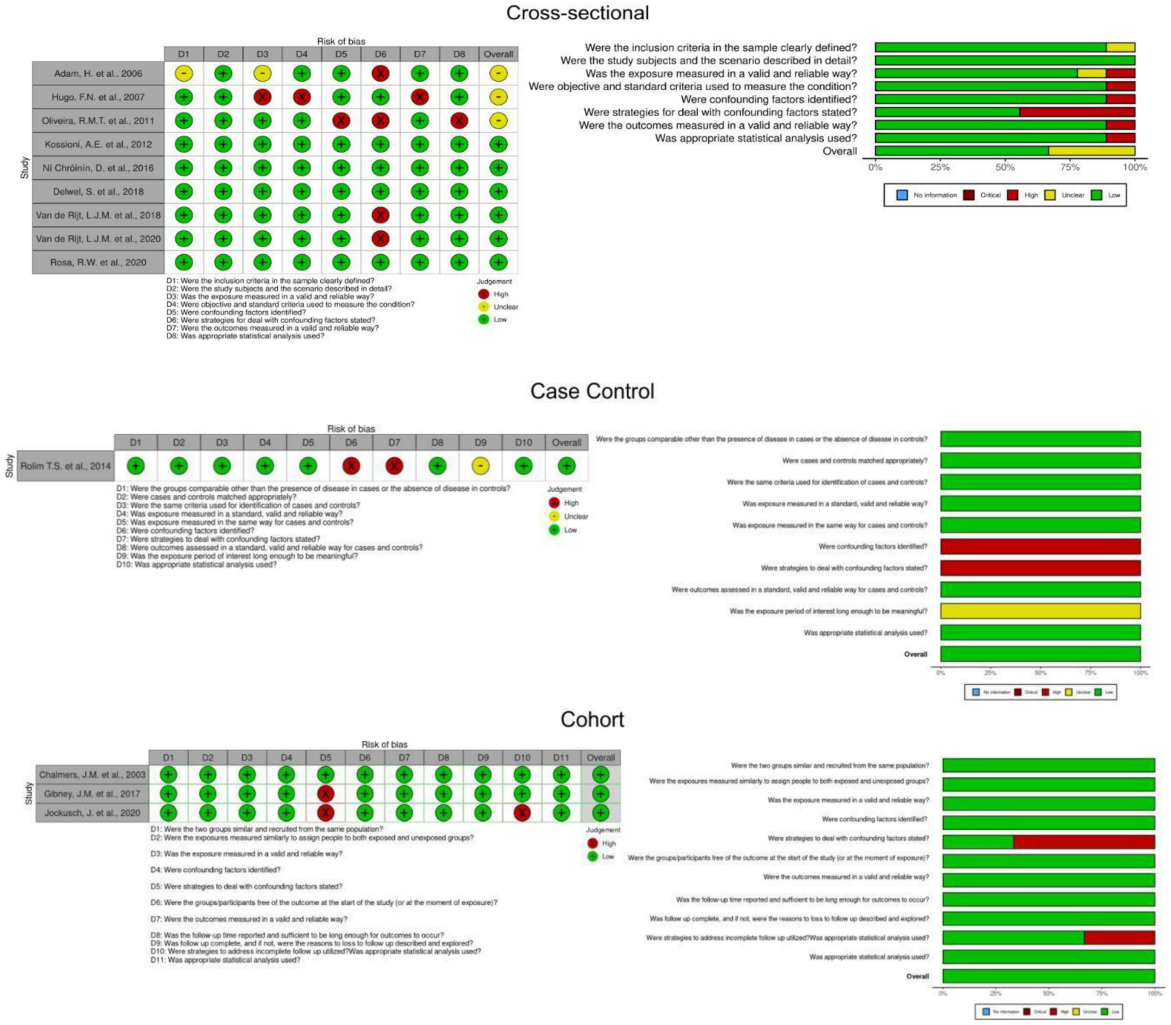
Risk of bias assessment for the studies included in the synthesis, evaluated using the JBI Critical Appraisal Tool. JBI, 2020. Green indicates a low risk of bias, yellow indicates a moderate risk of bias, and red indicates a high risk of bias

As shown in Figure 2, of the thirteen studies analyzed, three had a moderate risk of bias^(14-16)^, and ten had a low risk of bias^(3,4,17-24)^. The limitations of the studies included a lack of clarity in the strategies for identifying and addressing confounding factors^(3,4,16,20,23,24)^ as well as inclusion criteria^(14)^, whether the exposure was measured validly and reliably^(14,15)^, whether the criteria and outcomes were measured objectively and consistently^(15)^, and whether the statistical analysis was conducted validly and reliably^(16)^. Additional limitations were identified regarding whether the exposure period was sufficiently long to be significant^(3)^ and whether strategies were implemented to address incomplete follow-up results from patients who, for some reason, had to withdraw from the study^(24)^.

### Individual study results

Most studies did not identify significant differences in the prevalence of orofacial pain based on the specific type of dementia. However, it was observed that patients in more advanced stages, who had difficulty verbally reporting pain, were at a higher risk of experiencing^(18)^. Regarding gender, the studies indicated a higher prevalence among women in the population^(3,4,14-24)^.

The main factors linked to orofacial pain in patients with dementia include poor oral hygiene, having natural teeth, tooth pain, ulcers, and xerostomia^(3,4,14-24)^. Dentate patients with dementia, especially those with poor oral hygiene, are more likely to develop orofacial pain^(4)^. Additionally, patients with dementia showed higher rates of periodontal infections, cavities, and residual roots^(3,15-17,20,21)^.

The locations where these patients were assessed and evaluated varied, with four studies conducted in nursing homes^(4,16,19,24)^, five studies in hospitals^(3,16,17,19,22)^, three studies in outpatient clinics^(15,16,19)^, and two studies in community interventions^(21,22)^_._

### Synthesis of results

**Figure 3:**
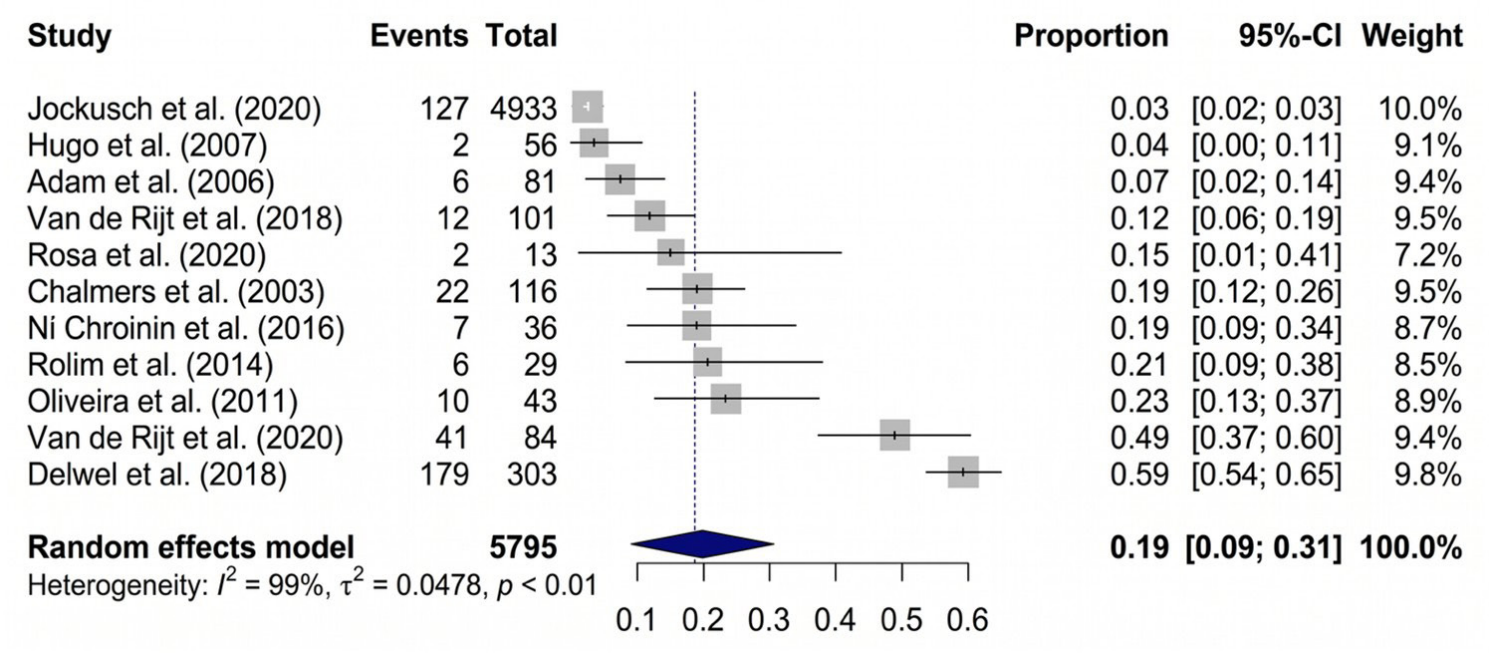
Forest plot of the prevalence of orofacial pain in older individuals with dementia

Eleven studies were included in the quantitative synthesis, which evaluated the prevalence of orofacial pain in patients with dementia. Specifically, regarding the prevalence of orofacial pain in older individuals with dementia (Figure 3), a rate of 19% [95% Confidence Interval = 9% - 31% I^2^=99%] was found, indicating a 95% chance that the true prevalence falls between 9% and 31%. The confidence interval shows the uncertainty about this estimate, and the high heterogeneity highlights significant variability among the studies analyzed. This indicates the need for further research to improve the estimate and investigate the causes of this variability. When data is missing, up to three attempts were made to contact four authors to request the missing information. Of these, two authors provided the necessary data^(18,21)^, one author did not respond to the contact attempts^(23)^, and another stated that the requested information was not available^(17)^.

## DISCUSSION

Orofacial pain in older individuals with dementia is not just physical discomfort but a complex interaction between cognitive decline and the inability to communicate suffering, which worsens overall health and significantly reduces quality of life for these individuals^(5,25,26)^. This study aimed to identify how common orofacial pain is among older adults with dementia through a systematic literature review. It was found that about 1 in every 5 older adults with dementia experiences some type of orofacial pain.

Aging is directly associated with an increased risk of cognitive decline, which can cause changes in mental abilities^(27)^. This decline in cognitive function impairs self-care and the ability to report complaints^(28)^. According to data from studies included in the meta-analysis, about 19% of older individuals with dementia experience orofacial pain. These findings highlight the importance of public policies aimed at this population. Given the communication difficulties faced by these patients, it is essential to provide more specialized care and train healthcare professionals to properly manage this condition.

Age is recognized as a risk factor for developing both dementia and pain. As people age, these conditions become more common, and it is projected that in the future, the number of affected individuals will increase even further, with a particularly high occurrence of dementia among older women. This rise is due to the higher prevalence of dementia in older women and their longer survival with the disease, especially in cases of Alzheimer’s^(26)^. The literature highlights that the combination of aging and dementia worsens these factors, making it more difficult to manage orofacial pain in this population. Cognitive decline linked to dementia hampers self-care, leading to a buildup of oral problems, which further increases the risk of orofacial pain^(8)^.

In the analyzed studies, there was a higher occurrence of odontogenic pain, including tooth pain, oral pain, and pain during oral hygiene^(14,15,18,19,22-24)^. However, there were also reports of pain suggestive of non-odontogenic origin, such as resting pain, headaches, and ulcers^(3,4,17,19,20)^. These findings suggest that most patients evaluated have poor oral health, indicating that odontogenic pain, associated with complaints and difficulties in chewing, may contribute to the development of non-odontogenic pain, such as temporomandibular disorders and myalgias.

From a biological perspective, the occurrence and recognition of orofacial pain in individuals with dementia can be explained by neurodegenerative changes that affect pain processing and communication abilities^(29)^. Dementia disrupts brain systems responsible for sensory, cognitive, and affective aspects of pain, which may alter pain perception and limit the capacity to express discomfort^(29)^. Cerebrovascular disease and white matter damage, frequently observed in Alzheimer’s disease and vascular dementia, may further impair neural connectivity and nociceptive integration, increasing vulnerability to pain while hindering its clinical recognition^(29)^. In parallel, chronic pain has been associated with structural and functional changes in cortical and subcortical regions involved in nociception and cognition, suggesting an interaction between persistent pain states and neurodegenerative processes^(30)^. From a peripheral standpoint, studies consistently demonstrate poorer oral health in people with dementia, with a higher prevalence of coronal and root caries, retained roots, and inadequate oral hygiene, which represent persistent sources of nociceptive stimulation in the orofacial region^(8,20)^. These conditions tend to worsen as cognitive decline compromises self-care and access to dental treatment, contributing to the maintenance of orofacial pain. Taken together, the interaction between central neurodegenerative alterations and peripheral oral pathology provides a plausible explanation for both the high prevalence and the frequent under-recognition of orofacial pain in individuals with dementia.

The patients evaluated were in nursing homes, hospitals, and outpatient clinics, under the supervision of senior caregivers and nurses^(3,4,14-20,23,24)^. A systematic review investigated how oral health education programs for caregivers affect the oral hygiene of older adults, highlighting that poor oral health in seniors can cause serious health problems, such as malnutrition and cognitive decline^(31)^. It was observed that educational programs significantly enhanced caregivers' knowledge and older adults' oral health, as shown by increased normal mucosa percentages, decreased visible plaque, and fewer cases of denture stomatitis after the programs were implemented^(31)^. These findings emphasize the need for ongoing education programs for caregivers and nurses in managing orofacial pain and preventing dental problems in patients with dementia. Teaching care givers proper oral hygiene practices is crucial to prevent more serious complications and to enhance the quality of life for these patients.

Although this study helps understand the prevalence of orofacial pain in patients with dementia, some limitations should be acknowledged. First, the number of studies available in the literature remains limited, which restricts the ability to generalize the results. However, a rigorous methodology was employed in selecting and analyzing studies, including higher-quality studies through a risk of bias assessment, to ensure the reliability of the information. Additionally, the heterogeneity of the studies makes it difficult to compare results, which directly increases variability in prevalence estimates. However, this diversity reflects the complexity of the situation and the need for a more comprehensive study, further emphasizing the importance of this work in addressing this gap in the literature. This variability may be further explained by several factors, including differences in assessment settings, such as hospitals, outpatient clinics, and nursing homes, as well as the inclusion of distinct categories of pain, namely odontogenic and non-odontogenic conditions. In addition, heterogeneity is likely influenced using different diagnostic approaches, including behavioral instruments such as the OPS-NVI. Importantly, the progressive cognitive decline characteristic of dementia substantially limits the capacity for self-reporting pain. As a result, pain assessment often relies on caregiver reports or observational measures, both of which are subject to interpretation and variability. This reliance may partially account for the inconsistencies observed across studies.

Another important limitation is the challenge of communicating pain in patients with dementia. Studies indicate that the Orofacial Pain Scale for Non-Verbal Individuals (OPS-NVI) is a valuable tool for evaluating pain in older patients with dementia. The scale relies on observing behaviors associated with orofacial pain in various situations, such as resting, drinking, chewing, and oral hygiene care. These behaviors include facial expressions, body movements, vocalizations, and specific actions, with pain intensity scored on a scale from 0 to 10. Therefore, behavioral observation is an effective alternative for this assessment^(4,19,20)^. However, another study using the OPS-NVI found low favorable concordance and high negative concordance rates, concluding that this scale is not recommended as a screening tool for orofacial pain^(32)^. This clinical discrepancy highlights a significant controversy regarding the instrument's reliability and underscores a broader challenge, as progressive communicative impairment in dementia prevents the use of traditional self-report, there is a clinical imperative for more effective and standardized assessment strategies. Given the high prevalence of orofacial pain identified in this meta-analysis, the current lack of consensus on screening tools necessitates the development and rigorous validation of behavioral instruments tailored for routine use by both caregivers and healthcare professionals. Enhancing the effectiveness of these tools through standardized protocols is essential to ensure that non-verbal signals are not overlooked, effectively bridging the gap between clinical necessity and accurate diagnosis in this vulnerable population.

Despite this, the reliability between evaluators and researchers can be improved when complementary strategies are adopted, highlighting the need for more integrated follow-up and analysis for these patients. Nonetheless, this study offers potential insights into the prevalence of orofacial pain in this vulnerable population, emphasizing the importance of policies targeting this group due to the significant prevalence of this issue.

## CONCLUSION

About one in five older individuals with dementia experience orofacial pain, highlighting a significant prevalence that can significantly impact their quality of life. Recognizing and managing this condition remains difficult mainly due to communication challenges inherent in these patients, emphasizing the need for specialized approaches to ensure effective and compassionate care. It is recommended to establish ongoing training programs for healthcare professionals and caregivers that focus on accurate diagnosis and management of oral health to prevent complications. Health policies should prioritize dental services in long-term care facilities and hospitals, ensuring access to preventive care and specialized treatments. Collaboration among dentists, geriatricians, neurologists, and psychologists should be promoted to create multidisciplinary teams for comprehensive management of orofacial pain in older adults with dementia. Additionally, awareness campaigns about the prevalence and signs of this pain should be encouraged, highlighting the importance of oral health for the quality of life of older adults. These efforts are essential for improving the quality of life for this vulnerable group and reducing the burden of orofacial pain on healthcare systems.

## Appendix 1 Reasons for study exclusion

**Table:**

**Author, Year**	**Reason for Exclusion**
Cohen-Mansfield, J., 2002	3
Hsu, K. T. et al., 2007	3
Van Kooten, J. et al., 2015	3
Ahmed, A. I. et al., 2016	3

Legend:

1. Studies whose population wasn't elderly were excluded.

2. Studies where individuals didn't have dementia, or where dementia wasn't proven by any assessment method were excluded.

3. Studies that didn't assess prevalence of orofacial pain, or when data were unavailable even after contact with the authors were excluded.

4. Studies that have not evaluated at least one of the outcomes of interest.

5. Reviews, books, expert opinions, in vitro studies, animal studies, letters to the editor, conference abstracts.

REFERENCE

1. Cohen-Mansfield J. Relatives' assessment of pain in cognitively impaired nursing home residents. J Pain Symptom Manage. 2002 Dec;24(6):562-71. doi: 10.1016/s0885-3924(02)00521-3. PMID: 12551805.

2. Hsu KT, Shuman SK, Hamamoto DT, Hodges JS, Feldt KS. The application of facial expressions to the assessment of orofacial pain in cognitively impaired older adults. J Am Dent Assoc. 2007 Jul;138(7):963-9; quiz 1021-2. doi: 10.14219/jada.archive.2007.0293. PMID: 17606495.

3. van Kooten J, Delwel S, Binnekade TT, Smalbrugge M, van der Wouden JC, Perez RS, Rhebergen D, Zuurmond WW, Stek ML, Lobbezoo F, Hertogh CM, Scherder EJ. Pain in dementia: prevalence and associated factors: protocol of a multidisciplinary study. BMC Geriatr. 2015 Mar 21;15:29. doi: 10.1186/s12877-015-0025-0. PMID: 25879681; PMCID: PMC4436741.

4. Isam Ali Ahmed A. Clinical pharmacology of oral tetrahydrocannabinol in older people with dementia [Doctoral Thesis]. Omdurman: Radboud University Nijmegen; 2016. 317 p. Available on: https://hdl.handle.net/2066/159495 ISBN 9789462287884

## Appendix 2 Database search strategy

**Table:**

**Electronic Databases**	Searches conducted on 06/26/2023 Update on 09/25/2025
**Embase** **151/40**	('Dementia' OR 'Dementias' OR 'Amentia' OR 'Amentias' OR 'Familial Dementia' OR 'Familial Dementias') AND ('Facial Pain' OR 'Face Pain' OR 'Orofacial Pain' OR 'Neuralgic Facial Pain' OR 'Craniofacial Pain' OR 'Myofacial Pain' OR 'Temporomandibular painful' OR 'Non Odontogenic Pain' OR 'Odontogenic Pain' OR 'Dentoalveolar Pain' OR 'Idiopathic Dentoalveolar Pain' OR 'Myofascial Pain Syndromes' OR 'Myofascial Pain Syndrome' OR 'Myofascial Trigger Point Pain')
**Cochrane** **10/2**	("Dementia" OR "Dementias" OR "Amentia" OR "Amentias" OR "Familial Dementia" OR "Familial Dementias") AND ("Facial Pain" OR "Face Pain" OR "Orofacial Pain" OR "Neuralgic Facial Pain" OR "Craniofacial Pain" OR "Myofacial Pain" OR "Temporomandibular Painful" OR "Non Odontogenic Pain" OR "Odontogenic Pain" OR "Dentoalveolar Pain" OR "Idiopathic Dentoalveolar Pain" OR "Myofascial Pain Syndromes" OR "Myofascial Pain Syndrome" OR "Myofascial Trigger Point Pain")
**Lilacs** **3/0**	("Demência" OR "Demência Senil Tipo Paranoide" OR " Dementia" OR "Amentia" OR "Amentias" OR "Dementia, Familial" OR "Dementias" OR "Familial Dementia" OR "Familial Dementias" OR "Senile Paranoid Dementia" OR "Senile Paranoid Dementias" OR "Demencia" OR "Demencia Paranoide Senil") AND ("Dor Facial" OR "Dor Craniofacial" OR "Dor Miofacial" OR "Dor Orofacial" OR "Facial Pain" OR "Craniofacial Pain" OR "Face Pain" OR "Myofacial Pain" OR "Neuralgic Facial Pain" OR "Orofacial Pain" OR "Dolor Facial" OR "Dolor Craneofacial" OR "Dolor Miofacial" OR "Dolor Orofacial" OR "Dor Temporomandibular" OR "Temporomandibular Painful" OR "Dolor Temporomandibular" OR "Dor não odontogênica" OR "Non Odontogenic Pain" OR "Dolor no Odontogénico" OR "Dor Odontogénica" OR "Odontogenic Pain" OR "Dolor Odontogénico" OR "Dor Dentoalveolar" OR "Dentoalveolar Pain" OR "Dolor dentoalveolar" OR "Dor Dentoalveolar Idiopática" OR "Idiopathic Dentoalveolar Pain" OR "Dolor Dentoalveolar Idiopático" OR "Síndromes da Dor Miofascial" OR "Síndromes del Dolor Miofascial")
**Livivo** **141/4**	("Dementia" OR "Dementias" OR "Amentia" OR "Amentias" OR "Familial Dementia" OR "Familial Dementias") AND ("Facial Pain" OR "Face Pain" OR "Orofacial Pain" OR "Neuralgic Facial Pain" OR "Craniofacial Pain" OR "Myofacial Pain" OR "Non Odontogenic Pain" OR "Odontogenic Pain" OR "Dentoalveolar Pain" OR "Idiopathic Dentoalveolar Pain" OR "Myofascial Pain Syndromes" OR "Myofascial Pain Syndrome" OR "Myofascial Trigger Point Pain")
**Pubmed/Medline** **54/19**	1. ("Dementia"[Mesh] OR "Dementia" OR "Dementias" OR "Amentia" OR "Amentias" OR "Familial Dementia" OR "Familial Dementias") 2. ("Facial Pain"[Mesh] OR "Face Pain" OR "Orofacial Pain" OR "Neuralgic Facial Pain" OR "Craniofacial Pain" OR "Myofascial Pain" OR "Myofascial Pain Syndromes"[Mesh] OR "Myofascial Pain Syndrome" OR "Myofascial Trigger Point Pain") #1 AND #2
**Scopus** **110/14**	TITLE-ABS-KEY("Dementia" OR "Dementias" OR "Amentia" OR "Amentias" OR "Familial Dementia" OR "Familial Dementias") AND TITLE-ABS-KEY ("Facial Pain" OR "Face Pain" OR "Orofacial Pain" OR "Neuralgic Facial Pain" OR "Craniofacial Pain" OR "Myofacial Pain" OR "Temporomandibular Painful" OR "Non Odontogenic Pain" OR "Odontogenic Pain" OR "Dentoalveolar Pain" OR "Idiopathic Dentoalveolar Pain" OR "Myofascial Pain Syndromes" OR "Myofascial Pain Syndrome" OR "Myofascial Trigger Point Pain")
**Web os Science** **60/19**	1.("Dementia" OR "Dementias" OR "Amentia" OR "Amentias" OR "Familial Dementia" OR "Familial Dementias") 2.("Facial Pain" OR "Face Pain" OR "Orofacial Pain" OR "Neuralgic Facial Pain" OR "Craniofacial Pain" OR "Myofacial Pain" OR "Temporomandibular Painful" OR "Non Odontogenic Pain" OR "Odontogenic Pain" OR "Dentoalveolar Pain" OR "Idiopathic Dentoalveolar Pain" OR "Myofascial Pain Syndromes" OR "Myofascial Pain Syndrome" OR "Myofascial Trigger Point Pain") 3. #1 AND #2
**Gray Literature**	
**Proquest** **10/0**	NOFT("Dementia" OR "Dementias" OR "Amentia" OR "Amentias" OR "Familial Dementia" OR "Familial Dementias") AND NOFT("Facial Pain" OR "Face Pain" OR "Orofacial Pain" OR "Neuralgic Facial Pain" OR "Craniofacial Pain" OR "Myofacial Pain" OR "Temporomandibular Painful" OR "Non Odontogenic Pain" OR "Odontogenic Pain" OR "Dentoalveolar Pain" OR "Idiopathic Dentoalveolar Pain" OR "Myofascial Pain Syndromes" OR "Myofascial Pain Syndrome" OR "Myofascial Trigger Point Pain")
**Google Acadêmico** **100/100**	(Dementia) AND (Facial Pain OR Orofacial Pain)filetype:pdf

## Appendix 3 Characteristics of included studies

**Table 1:** Characteristics Included Studies.

**Author, Year, and Country**	**Study Design**	**Sample Gender**	**Population**	**Age (mean or range)**	**Dementia (%)**	**Pain (%)**	**Type of Pain**
Chalmers et al., 2003 (Australia)	Cohort	n=232 (98F/134M)	Older	≤79: 78.4% ≥80: 21.6%	116 (50%)	22 (19%)	Tooth Pain
Adam et al., 2006 (United Kingdom)	Cross-sectional	n=135 (93F/42M)	Older	65 to 100	81 (60%)	6 (7.4%)	Oral pain
Hugo et al., 2007 (Brazil)	Cross-sectional	n=56 (30F/26M)	Older	76.09 (±7.76)	56 (100%)	2 (3.6%)	Oral pain, during oral hygiene
Oliveira et al., 2011 (Brazil)	Cross-sectional	n=70 (51F/19M)	Older	58 to 95	43 (61.4%)	10 (23.26%)	Oral pain
Kossioni et al., 2012 (Greece)	Cross-sectional	n=111 (70F/41M)	Older	57 to 94	27 (24.3%)	**	Ulcerations while chewing
Rolim TS, et al 2014 (Brazil)	Case-control	n= 59	Older	59 to 91	29 (49%)	6 (20.7%)	Sleep bruxism, Biting, Chewing, Generalized pain, Headache
		(38 F/21M)					
Ní Chróinín et al., 2016 (Australia)	Cross-sectional	n=202 (124F/78M)	Older	≥70 (84.4±6.5)	36 (17.8%)	7(19.44%)	Tooth pain
Gibney et al., 2017 (Australia)	Cohort	n=575 (343F/232M)	Older	65 to 96+	161 (28.0%)	**	Tooth pain
Delwel S. et al., 2018(Netherlands)	Cross-sectional	n=348	Older	66 to 102	303 (87.1%)	179 (25.7%)	At rest, Chewing, Oral hygiene care, Provoked sensory
		(230 F/118M)					
Van de Rijt, et al., 2018(United Kingdom)	Cross-sectional	n=101 (70F/31M)	Older	70 to 99	101 (100%)	33 (32.6%)	Resting, Chewing
Van de Rijt, et al, 2020 (United Kingdom)	Cross-sectional	n=111 (69=F/42=M)	Older	65 to 101	29 (49%)	37(48.8%)	Resting, Drinking, Chewing, During oral hygiene
Jockusch et al., 2020 (Switzerland)	Cohort	n=7,922 (5,903F/2,019M)	Older	82.6 ± 8.9 (moderate dementia) 80.8 ± 8.9 (severe dementia)	4,933 (62.66%)	126.85 (2.57%)	Oral pain
Rosa et al., 2020 (France)	Cross-sectional	n=90 (68=F/22=M)	Older	91 to 106	13(15%)	2(15%)	Oral pain

**Legend:** F: Female, M: Male; ** Not reported

REFERENCE

1. Chalmers JM, Carter KD, Spencer AJ. Oral diseases and conditions in community-living older adults with and without dementia. Spec Care Dentist. 2003;23(1):7-17. doi: 10.1111/j.1754-4505.2003.tb00283.x. PMID: 12887148.

2. Adam H, Preston AJ. The oral health of individuals with dementia in nursing homes. Gerodontology. 2006 Jun;23(2):99-105. doi: 10.1111/j.1741-2358.2006.00118.x. PMID: 16677183.

3. Hugo FN, Hilgert JB, Bertuzzi D, Padilha DM, De Marchi RJ. Oral health behaviour and socio-demographic profile of subjects with Alzheimer's disease as reported by their family caregivers. Gerodontology. 2007 Mar;24(1):36-40. doi: 10.1111/j.1741-2358.2007.00149.x. PMID: 17302929.

4. Oliveira RM, Lia EM, Macedo SB, Amorim RF. Status da saúde bucal em pacientes com demência senil. ROBRAC: Rev Odontol Bras Central. 2011 Jan;20:114-

5. Kossioni AE, Kossionis GE, Polychronopoulou A. Oral health status of older hospitalised psychiatric patients. Gerodontology. 2012 Dec;29(4):272-83. doi: 10.1111/j.1741-2358.2012.00633.x. Epub 2012 Mar 1. PMID: 22380633.

6. de Souza Rolim T, Fabri GM, Nitrini R, Anghinah R, Teixeira MJ, de Siqueira JT, Cestari JA, de Siqueira SR. Oral infections and orofacial pain in Alzheimer's disease: a case-control study. J Alzheimers Dis. 2014;38(4):823-9. doi: 10.3233/JAD-131283. PMID: 24077432.

7. Ní Chróinín D, Montalto A, Jahromi S, Ingham N, Beveridge A, Foltyn P. Oral Health Status Is Associated with Common Medical Comorbidities in Older Hospital Inpatients. J Am Geriatr Soc. 2016;64(8):1696-700. doi: 10.1111/jgs.14247. PMID: 27487009.

8. Gibney JM, Wright C, Sharma A, D'Souza M, Naganathan V. The oral health status of older patients in acute care on admission and Day 7 in two Australian hospitals. Age Ageing. 2017 ;46(5):852-6. doi: 10.1093/ageing/afx085. PMID: 28541372.

9. Delwel S, Scherder EJA, de Baat C, Binnekade TT, van der Wouden JC, Hertogh CMPM, Maier AB, Perez RSGM, Lobbezoo

F. Orofacial pain and its potential oral causes in older people with mild cognitive impairment or dementia. J Oral Rehabil. 2019;46(1):23-32. doi: 10.1111/joor.12724. PMID: 30281826;

10. van de Rijt LJM, Weijenberg RAF, Feast AR, Vickerstaff V, Lobbezoo F, Sampson EL. Oral health and orofacial pain in people with dementia admitted to acute hospital wards: observational cohort study. BMC Geriatr. 2018;18(1):121. doi: 10.1186/s12877-018-0810-7. PMID: 29792172;

11. van de Rijt LJ, Feast AR, Vickerstaff V, Lobbezoo F, Sampson EL. Prevalence and associations of orofacial pain and oral health factors in nursing home residents with and without dementia. Age Ageing. 2020;49(3):418-24. doi: 10.1093/ageing/afz169. PMID: 31860004.

12. Rosa RW, Samot J, Helmer C, Pourtau G, Dupuis V, Fricain JC, Georget A, Dartigues JF, Arrivé E. Important oral care needs of older French people: A cross-sectional study. Rev Epidemiol Sante Publique. 2020;68(2):83-90. doi: 10.1016/j.respe.2020.01.135. PMID: 32111348.

13. Jockusch J, Riese F, Theill N, Sobotta BAJ, Nitschke I. Aspects of oral health and dementia among Swiss nursing home residents. Z Gerontol Geriatr. 2021;54(5):500-6. English. doi: 10.1007/s00391-020-01739-w. PMID: 32488304.
